# Integrated Single‐Myofibre Multi‐Omics Correlates Inflammatory State to Myosin Energetics in ICU‐Acquired Weakness

**DOI:** 10.1002/jcsm.70407

**Published:** 2026-10-19

**Authors:** Alexandra M. Winant, Roger Moreno‐Justicia, Lucia Paolini, Wout J. Claassen, Coen A. C. Ottenheijm, Atul S. Deshmukh, Stefano Cattaneo, Simone Piva, Nicola Latronico, Robert A. E. Seaborne, Julien Ochala

**Affiliations:** ^1^ Department of Biomedical Sciences, Faculty of Health and Medical Sciences University of Copenhagen Copenhagen Denmark; ^2^ Novo Nordisk Foundation Center for Basic Metabolic Research, Faculty of Health and Medical Sciences University of Copenhagen Copenhagen Denmark; ^3^ Department of Medical and Surgical Specialties, Radiological Sciences and Public Health Università di Brescia Brescia Italy; ^4^ Amsterdam UMC, Location VUmc Department of Physiology Amsterdam the Netherlands; ^5^ Department of Cellular and Molecular Medicine University of Arizona Tucson Arizona USA; ^6^ ASST Spedali Civili di Brescia, ASST Spedali Civili Dept. of Bone and Joint Surgery Brescia Italy; ^7^ Department of Anesthesia, Critical Care and Emergency ASST Spedali Civili University Hospital Brescia Italy; ^8^ Centre of Human and Applied Physiological Sciences, School of Basic and Medical Biosciences, Faculty of Life Sciences & Medicine King's College London London UK

**Keywords:** critical illness, multi‐omics, muscle weakness, myosin energetics, single‐fibre analysis, skeletal muscle heterogeneity

## Abstract

**Background:**

Skeletal muscle atrophy is a pervasive complication of critical illness that worsens survival and recovery, yet remains poorly explained by current clinical or molecular markers.

**Methods:**

To directly connect disease‐associated molecular states to the contractile machinery, this study combined sequential functional, transcriptomic and proteomic profiling of the same single human skeletal myofibres from critically ill patients in the intensive care unit with acquired weakness (ICU‐AW) and controls. This integrated approach provides a framework for validating molecular signals directly within the cellular and biomechanical context.

**Results:**

Differential expression analyses between control and ICU‐AW myofibres show marked donor‐level heterogeneity, with no differentially expressed features (FDR < 0.05). Ranked gene set enrichment analyses revealed distinct transcriptional and proteomic signatures between conditions, as well as a subtle yet conserved myofibre state enriched in ICU‐AW, characterised by inflammatory and chemotactic gene programmes, intracellular structural remodelling and bioenergetic adaptation. Nineteen features were significantly (FDR < 0.05) altered at both RNA and protein levels from the same myofibre. Overall, these 19 overlapping features link an inflammatory transcriptional landscape to a proteomic shift toward mitochondrial and translational machinery, revealing a coordinated stress‐adaptation programme with selective post‐translational control in fibres from patients with ICU‐AW. Further integration with the functional dataset revealed fibres in this state displayed selectively disrupted myosin dynamics, evidenced by prolonged ATP turnover time of myosin heads in their super‐relaxed conformation, suggesting altered myosin energetics as a contributor to muscle dysfunction.

**Conclusions:**

These findings define a discrete, disease‐associated myofibre state and validate a proof‐of‐concept integrative single‐fibre framework for connecting multi‐omic heterogeneity to molecular motor function in complex human disease. The identification of coordinated transcriptomic and proteomic programmes, validated through functional measurement, suggests that targeting this fibre state may offer new therapeutic opportunities in ICU‐acquired dysfunction and related muscle disorders.

## 1 Introduction

Skeletal muscle is a central determinant of whole‐body health, contributing to locomotion, posture, metabolic homeostasis and endocrine signalling throughout the lifespan. The vulnerability of skeletal muscle is particularly evident in critical illness, where patients may lose nearly 2% of muscle mass per day during the first week of intensive care unit (ICU) admission [1]. This rapid, and often profound, ICU‐acquired weakness (ICU‐AW) is incompletely explained by the admission diagnosis, treatment protocols or broad measures of muscle atrophy [2]. Loss of muscle mass or function can occur rapidly and is associated with impaired mobility, metabolic dysfunction, prolonged hospitalisation, and increased mortality across aging, chronic disease and critical illness [3, 4]. Hence, skeletal muscle maintenance is crucial and depends on coordinated regulation of multiple cell types and cell states within a structurally and molecularly complex tissue microenvironment [5].

Myofibres are the most abundant cell type in skeletal muscle and are classically categorised by myosin heavy chain isoform composition and metabolic properties [5]. While this classification captures key aspects of contractile specialisation, it underestimates the molecular diversity within and between individual myofibres [6]. As multinucleated syncytia, myofibres exhibit spatially organised and non‐uniform regulation along their length [7]. Muscle tissue homeostasis is further shaped by satellite cells, fibroblasts, endothelial cells and immune cells that collectively support tissue structure and function [5]. The resulting cellular and molecular heterogeneity of the muscle niche poses a challenge for achieving high‐resolution, cell‐specific measurements in human disease [8].

Mechanistic studies into ICU‐AW show a preferential loss of myosin and myosin‐associated proteins and a consistent reduction in myosin‐to‐actin ratios [9, 10, 11, 12, 13, 14]. There is increased inflammation and cytokine production, altered calcium homeostasis, enhanced proteolysis and bioenergetic failure as contributors to ICU‐acquired muscle dysfunction [15]. However, how these processes converge at the level of individual myofibres remains incompletely understood.

Myosin molecules dynamically cycle between biochemical states with distinct energetic properties, including the energy‐sparing super‐relaxed (SRX) and more active disordered‐relaxed (DRX) conformations, which together contribute to basal ATP turnover time and energetic demand in resting muscle [16]. Disruption of the balance between myosin states can impair muscle bioenergetics and contractile capacity even in the absence of altered muscle structure, suggesting that altered myosin dynamics might link molecular stress responses to functional weakness during critical illness [16]. Yet, how these energetic states are regulated within individual human myofibres in the setting of systemic inflammation, oxidative stress and mitochondrial dysfunction is largely unexplored [17].

Multiple transcriptomic [10, 11, 18, 19, 20, 21] and other high‐throughput [22, 23, 24] studies have mapped molecular changes in skeletal muscle during ICU‐AW, highlighting perturbed signalling pathways and altered gene expression programmes. However, transcriptional changes do not consistently translate into protein‐level or functional alterations, while bulk analyses obscure cell‐ and state‐specific behaviours of individual myofibres. Prior work has shown that single‐myofibre analyses can reveal physiologically relevant diversity in protein expression and function that is substantially reduced in monogenic myopathies, but these approaches typically capture only one molecular layer per fibre [6]. Here, building upon our recent work [25], an integrated single‐fibre approach was applied that combined myosin functional measurements with transcriptomic and proteomic profiling from the same human myofibre to define molecular states associated with ICU‐AW. By linking gene expression, protein abundance and myosin SRX/DRX dynamics within individual fibres, this study identified a reproducible, disease‐enriched myofibre state characterised by inflammatory signalling, intracellular structural remodelling and bioenergetic adaptation. The presence of this state across ICU‐AW patients supports a fibre‐specific molecular and functional programme induced by critical illness. This proof‐of‐concept study introduces an integrated approach as a framework for validating molecular signals directly within the cellular and biomechanical context.

## 2 Methods

### 2.1 Study Approval

Here, investigations only included human tissue. All clinical information and materials were obtained for diagnostic purposes with informed consent, conforming to the Declaration of Helsinki and Good Clinical Practice. The study was approved by the Ethics Committee of Brescia (Protocol number 3468). Additional biopsies were collected in the context of a single‐blinded, randomised controlled trial (RCT), which was discontinued due to low inclusion rates. The protocol has been filed on 27 July 2017 in the Clinical Trial Register under #NCT03231540 and was approved by the Medical Ethical Committee of VU Medical Center, Amsterdam, The Netherlands.

### 2.2 Human Participants, Tissue Collection and Storage

Skeletal muscle tissue biopsies were obtained from 16 subjects hospitalised in the Intensive Care Unit (ICU‐AW group, *n* = 8) and Orthopaedic ward (control donors, *n* = 8) of the Spedali Civili University Hospital in Brescia, Italy and from VU Medical Center, Amsterdam, Netherlands. All samples were analysed anonymously. The subjects were both male and female, with an age range of 19–88 years (average age 60 y/o). Individual ages are reported in Table S1. Human skeletal muscle tissue was dissected from quadriceps femoris, described in detail in the Supporting Information. The muscle samples harvested from patients hospitalised in ICU were obtained with a surgical biopsy, 8–10 days after ICU admission. The samples obtained from patients in the Orthopaedic Ward were obtained during total hip replacement performed for hip arthritis through a direct anterior approach.

### 2.3 Single Myofibre Isolation and Preparation

On the day of functional profiling, muscle samples were thawed briefly and transferred to cold relaxing buffer (composition: 4 mM Mg‐ATP, 1 mM free Mg^2+^, 10^−9^ μM free Ca^2+^, 20 mM imidazole, 7 mM EGTA, 14.5 mM creatine phosphate and KCl to adjust ionic strength to 180 mM; pH 7.0). Working rapidly to minimise RNA and protein degradation, individual myofibres were manually isolated under a dissecting microscope.

Individual myofibres were positioned on custom‐prepared microscopy slides fitted with half‐split copper transmission electron microscopy grids. Clamped myofibres were maintained on ice during isolation. Once all myofibres were clamped, the unclamped tail segments were carefully excised and placed into microtubes containing RLT + buffer. The spatial location of each fibre tail was meticulously recorded for later integration with functional data. Lysates were stored at −80°C pending RNA and protein isolation.

### 2.4 Mant‐ATP Chase Experiments for Myosin ATP Turnover Kinetics

Clamped myofibres on slides were permeabilised with 0.1% Triton X‐100 in relaxing buffer for 10 min at room temperature. A coverslip was then positioned over the grid and myofibres to form a flow chamber. The slide was incubated for 5 min in rigour buffer (composition: 120 mM potassium acetate, 5 mM magnesium acetate, 2.5 mM K_2_HPO_4_, 50 mM MOPS, 2 mM DTT; pH 6.8). The solution was then exchanged for rigour buffer containing 250 μM Mant‐ATP and incubated in darkness for an additional 5 min to label myosin heads.

After labeling, the slide was rapidly flushed with rigour buffer containing 4 mM ATP to initiate the ATP chase. Simultaneously, fluorescence decay was captured using an inverted Zeiss Axio Observer microscope. Two reference images were acquired immediately before ATP addition. Subsequent images were recorded every 5 s for a total of 5 min. A bright field image was acquired to show myofibre and sarcomeric structure (Image S1).

Fluorescence intensity within individual myofibres was analysed using ImageJ. For each frame, mean background fluorescence intensity was subtracted from the fibre‐specific fluorescence intensity. All intensities were normalised to the final Mant‐ATP image before washout.

The normalised fluorescence decay curves were fit to an unconstrained double exponential model:

$$\text{Normalised}\;\text{fluorescence}=1-P_{1}\left(1-\exp^{-\frac{t}{T_{1}}}\right)-P_{2}\left(1-\exp^{-\frac{t}{T_{2}}}\right)$$

In this model: P_1_ = amplitude of the initial rapid decay (disordered‐relaxed myosin heads); T_1_ = time constant of the fast decay phase (DRX ATP turnover rate); P_2_ = amplitude of the slower second decay (super‐relaxed myosin heads); T_2_ = time constant of the slow decay phase (SRX ATP turnover rate); Theoretical ATP consumption was solely calculated from these parameters [26].

Outliers were assessed with the ROUT method, no outliers were detected and fibres were not excluded.

### 2.5 RNA Isolation and Sequencing Library Preparation

mRNA was captured from 10 μL of fibre lysate using the NEBNext Poly(A) mRNA Magnetic Isolation Module according to the manufacturer's instructions. The resulting supernatant was retained for protein precipitation.

Captured mRNA was converted into Illumina‐compatible cDNA libraries using the NEBNext Single Cell/Low Input RNA Library Prep Kit. Reverse transcription and template switching were performed, followed by PCR amplification for 14 cycles (in cases of very low concentration, four additional PCR cycles were performed). Library quality was assessed on an Agilent TapeStation 4200, and concentration was determined using a Thermo Fisher QubitFlex fluorometer.

Per sample, 2 ng of cDNA was fragmented, end‐repaired and ligated with Illumina adapters. Libraries were amplified for an additional eight PCR cycles with unique NEBNext Multiplex Oligos. Fragment size and molarity were assessed on the TapeStation. Libraries were pooled at equimolar concentration (2 nM) and sequenced on a NovaSeq X Plus Series platform to generate 150 bp paired‐end reads.

### 2.6 Primary RNA‐Seq Data Processing

Raw RNA sequencing data were quality‐controlled and aligned using the nf‐core/rnaseq pipeline (version 3.19.0). Prior to pipeline execution, reads were trimmed using Flexbar according to recommendations from New England Biolabs. Alignment was performed with STAR, and transcript abundance was quantified with Salmon. Output count matrices were used for downstream analysis.

### 2.7 Sample Preparation for Proteomics Analyses

The supernatant from mRNA isolation was retained for protein analysis, performed as previously described [25]. Detailed description can be found in the Supporting Information. Protein was precipitated with 100% acetone in 100 mM Tris–HCl, digested with trypsin and Lys‐C, desalted and transferred to Evotips prior to LC–MS/MS analysis.

LC–MS/MS analyses were performed as previously described, and described further in the Supporting Information [25].

### 2.8 Primary MS Data Processing

MS spectra were processed with DIA‐NN software (version 2.0) [27], using a myofibre‐specific library developed in‐house [6]. Protein group quantification was based exclusively on proteotypic peptides, with the neural network configured to operate in double‐pass mode. The quantification strategy was set to ‘Robust LC (high accuracy)’, and match‐between‐runs was activated. All other parameters were left at default values, including a peptide length window of 7–30 amino acids and a precursor‐level FDR cutoff of 1%. Downstream bioinformatics were conducted using the PG_matrix file from the DIA‐NN output.

### 2.9 Transcriptomic Analysis

All bioinformatics analyses were performed in R (version 4.4.2). Raw transcriptomic count matrices and sample metadata were converted into a Seurat object (version 5.3.0) [28] for downstream analysis and then converted to a Single Cell Experiment (version 1.28.0) object for quality control and normalization. Specific downstream transcriptomic analyses are described in the Supporting Information.

### 2.10 Proteomic Processing and Analysis

Proteomic data (protein intensity matrix) were log_2_‐transformed. Samples with more than 50% missingness were filtered out. Proteins were retained if quantified in at least 70% of samples within each condition. Between‐sample normalisation was performed using the scale method in limma. Missing values were imputed using the tImpute() function from the PhosR package (version 1.16.0). PCA assessment indicated minimal batch‐driven variance; thus no correction was applied. Downstream analyses are described in the Supporting Information.

### 2.11 Multi‐Omic Integration

DEGs and DEPs were intersected based on shared gene symbols. Directional agreement was assessed by comparing the signs of fold changes. Overlapping features (*n* = 19) were extracted from the significantly changed RNA and protein expression matrices. For each fibre, average expression values across the 19 overlapping features were computed separately for RNA and protein data. These averages were z‐score normalised and averaged to generate a single composite RNA–protein score per fibre.

### 2.12 Functional Correlation Analysis

Associations between the composite score and functional measurements (P1, T1, P2, T2, and theoretical ATP turnover time) were tested using non‐parametric Wilcoxon rank‐sum tests. Linear regression was used to estimate the relationship between the composite score and functional parameters.

### 2.13 Quantification and Statistical Analysis

All reported *p*‐values for differential expression are FDR‐adjusted. FDR was controlled using the Benjamini‐Hochberg method. Differential expression: FDR < 0.05 was used as the significance threshold for DEGs and DEPs. Pathway enrichment analyses used FDR adjustment. Functional associations: Associations between the composite score and functional measurements were tested using Wilcoxon rank‐sum tests with two‐tailed p‐values. Significance threshold: *p* < 0.05.

Sample sizes: Transcriptomic analysis included 165 individual fibres (control *n* = 83, ICU‐AW *n* = 82) from 16 donors. Proteomic analysis included 124 fibres (control *n* = 62, ICU‐AW *n* = 62) from 16 donors. Cluster‐versus‐non‐cluster analysis included 117 fibres with both datasets (ICU‐AW‐cluster *n* = 32, non‐cluster ICU‐AW *n* = 17, control non‐cluster *n* = 68).

### 2.14 Availability of Data and Material

All the data analysed are presented here in the figures, additional files and public data repositories. Specifically, RNA‐sequencing data have been deposited in the European Genome‐phenome Archive under study ID EGAS50000001649. The mass spectrometry proteomics data have been deposited to the ProteomeXchange Consortium via the PRIDE partner repository with the dataset identifier PXD073993. Custom R scripts for data processing, analysis, visualisation and statistical testing are available on GitHub: (https://github.com/amwinant/ICUAW‐SFMO‐pilot.git).

## 3 Results

### 3.1 Workflow Integrates Single‐Fibre Transcriptomics, Proteomics and Myosin Dynamics

Originally, we developed a novel workflow enabling sequential isolation and analysis of the same myofibre across multiple molecular and functional layers. Individual myofibres from the quadriceps femoris of eight ICU‐AW patients and eight control donors (orthopaedic surgery patients) were manually isolated and divided into two segments (Table 1). The first segment underwent Mant‐ATP chase experiments to measure myosin ATP turnover kinetics (SRX and DRX states); the second segment was processed for simultaneous transcriptomic and proteomic analysis, with careful registration to enable later dataset integration.

**TABLE 1:** Donor characteristics.

Characteristic	Case *N* = 8	Control *N* = 8
Sex, *N* (%)		
Male	7 (87.5%)	4 (50%)
Female	1 (12.5%)	4 (50%)
Age, arithmetic mean	60	64
Country of origin		
Netherlands	4 (50%)	0 (0%)
Italy	4 (50%)	8 (100%)
Reason for admission, *N* (%)		
Sepsis or septic shock	2 (25%)	0 (0%)
ARDS	4 (50%)	0 (0%)
Trauma	2 (25%)	8 (100%)
Days between ICU admission and biopsy		
6	1 (12.5%)	—
7	3 (37.5%)	—
8	2 (25%)	
12	1 (12.5%)	—
13	1 (12.5%)	—

For each donor, 12 myofibres were initially isolated and processed through both functional and multi‐omic pipelines. Transcriptomic libraries were prepared with individual molecular barcodes enabling pooled sequencing. Quality control filtering removed transcriptomic samples with gene counts or feature abundance ±3 median absolute deviation (MAD), and proteomic samples with more than 50% missingness (Figure 1a,b). This resulted in 165 fibres passing transcriptomic quality control (QC) and 124 passing proteomic QC, with 117 fibres common to both datasets (Figure 1c). Transcriptomic sequencing achieved an average depth of 5.7 × 10^6^ reads per sample with 13 456 ± 2682 unique genes detected. Proteomic analysis detected 1372 ± 70 proteins per sample with 8% average missingness, after filtering out proteins not present in at least 70% of samples from the same condition (Figure 1d). This depth and coverage highlight that our single‐fibre, multi‐omic approach has sufficient resolution for detecting both common and subtle molecular features in human skeletal muscle samples (Figure 1e). Workflow and successful number of fibres analysed per subject are presented in Table S1. PCA plots for RNA and protein on fibre‐ and donor‐level are presented in Figure S1.

**FIGURE 1:**
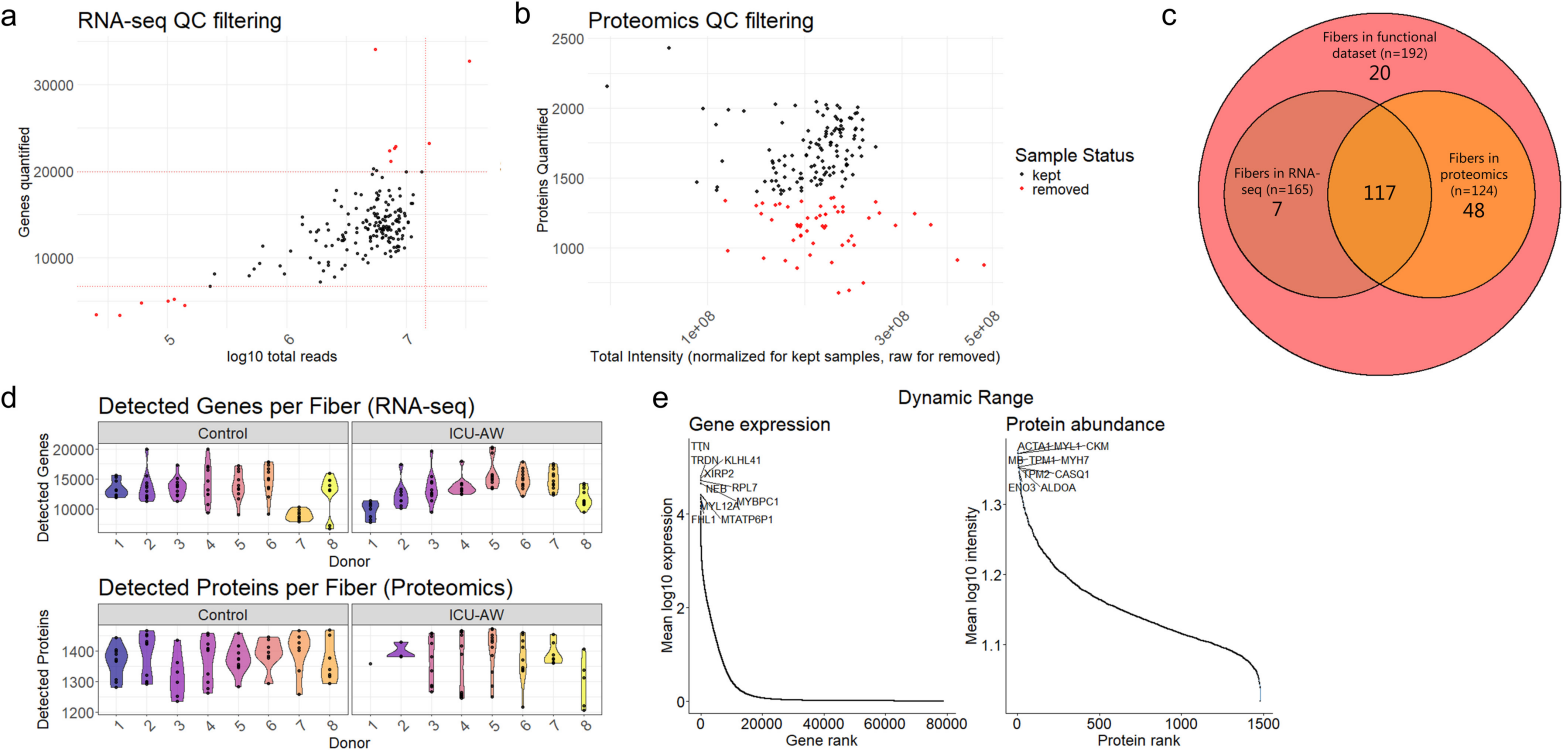
Quality control metrics by dataset. (a) Filtering of RNA‐seq data. Red dotted lines mark three median absolute deviations. Samples outside this cutoff were excluded (red dots), while black dots show retained samples. (b) Filtering of proteomic samples. Total intensities were normalised across kept samples (≤ 50% sample, ≤ 70% protein missingness). Samples failing filters are plotted in grey using raw intensity to show QC status. (c) Overlap of retained samples after filtering. (d) Detected features per fibre. Violin plots show detected features per fibre for RNA (top) and protein (bottom). (e) Dynamic range of mRNA and protein abundance. Genes (RNA) and proteins were ranked by their mean abundance across fibres following log‐transformation.

### 3.2 Subset of ICU‐AW Myofibres Display Distinct Inflammatory and Stress‐Response Gene Programmes Without Genome‐Wide Expression Shifts

Differential expression analysis was performed on transcriptomic data pseudobulked by donor to account for nested donor structure (donor‐level comparison). Although no differentially expressed genes (DEGs; FDR < 0.05) were identified when directly comparing control and ICU‐AW groups (Figure 2a), ranked gene set enrichment analysis using the fgsea algorithm with 10 000 permutations (GSEA; Hallmark pathways) revealed distinct higher‐level transcriptional signatures between conditions (Figure 2b). Fibres from ICU‐AW patients were characterised by upregulation of stress‐ and inflammation‐related pathways including reactive oxygen species pathway, protein secretion, IL6‐JAK‐STAT3 signalling, MYC targets and DNA repair. Conversely, pathways related to metabolism, immune homeostasis and tissue maintenance (including adipogenesis, myogenesis and interferon responses) were downregulated in fibres from ICU‐AW patients (Figure S2).

**FIGURE 2:**
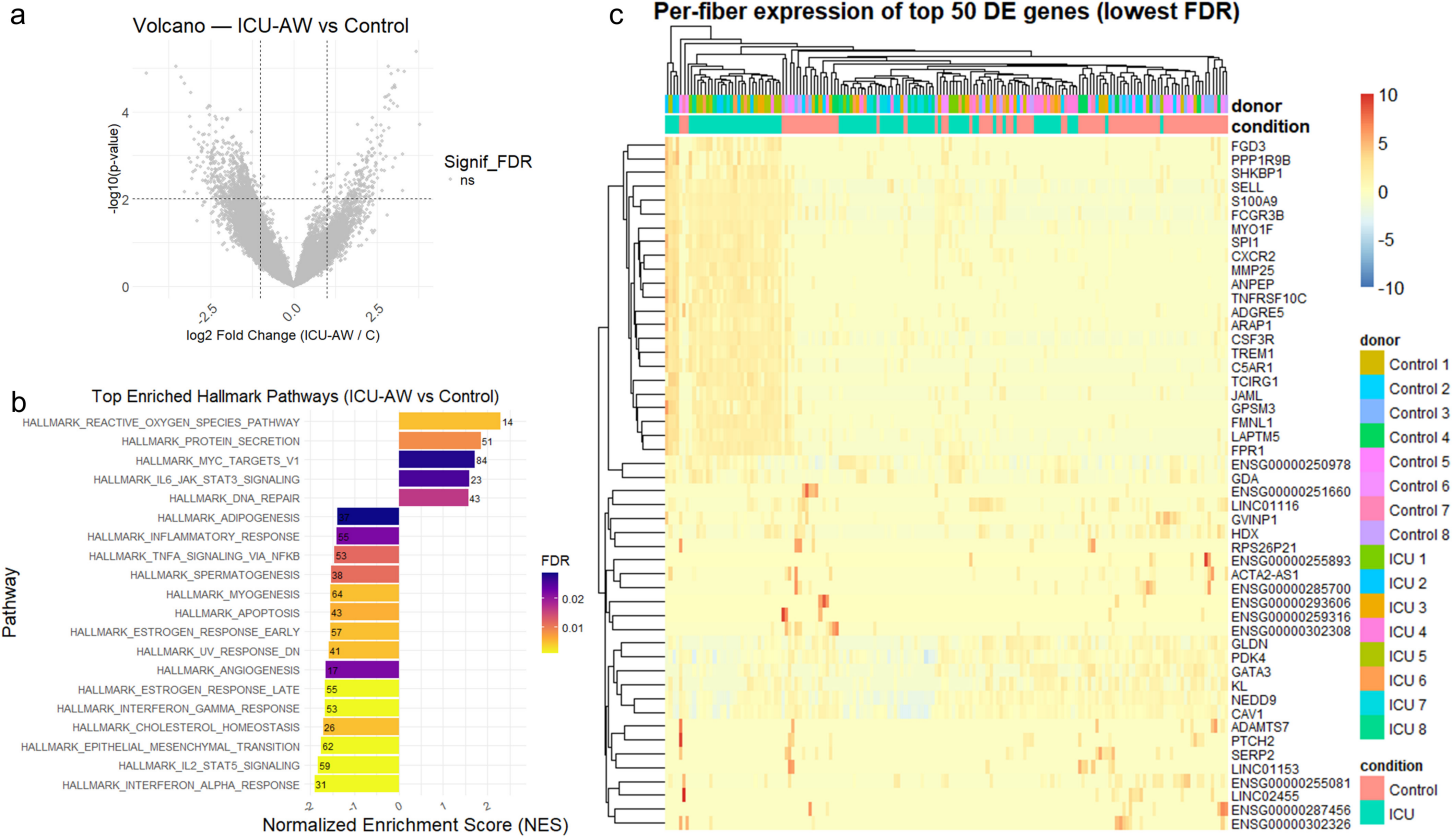
Transcriptional alterations in single myofibres associated with ICU‐AW. (a) Volcano plot of DEGs between ICU‐AW and control samples. Fibres pseudobulked by donor. Grey dots indicate non‐significant genes. (b) Hallmark pathway enrichment analysis for ICU‐AW. The top 20 significantly enriched pathways are ranked by normalised enrichment score (NES) and coloured by false discovery rate (FDR). Numbers adjacent to each bar indicate the leading‐edge gene count. (c) Heatmap of top 50 genes (by lowest FDR) from pseudobulk DE analysis on single fibres.

Hierarchical clustering of the top 50 DEGs (by FDR from donor‐level analysis) revealed variation in gene expression patterns across individual fibres (Figure 2c). A distinct subcluster emerged with consistently elevated expression of the top 23 DEGs. Strikingly, this subcluster was predominantly composed of myofibres from ICU‐AW patients (*n* = 32) with only one control fibre, suggesting a conserved transcriptional phenotype within the ICU‐AW population (Figure 3a). The subcluster is not defined by fibre type (with similar myosin heavy chain composition to the general dataset) or feature counts (Figure S3). This subcluster was designated the ‘Inflammatory’ cluster.

**FIGURE 3:**
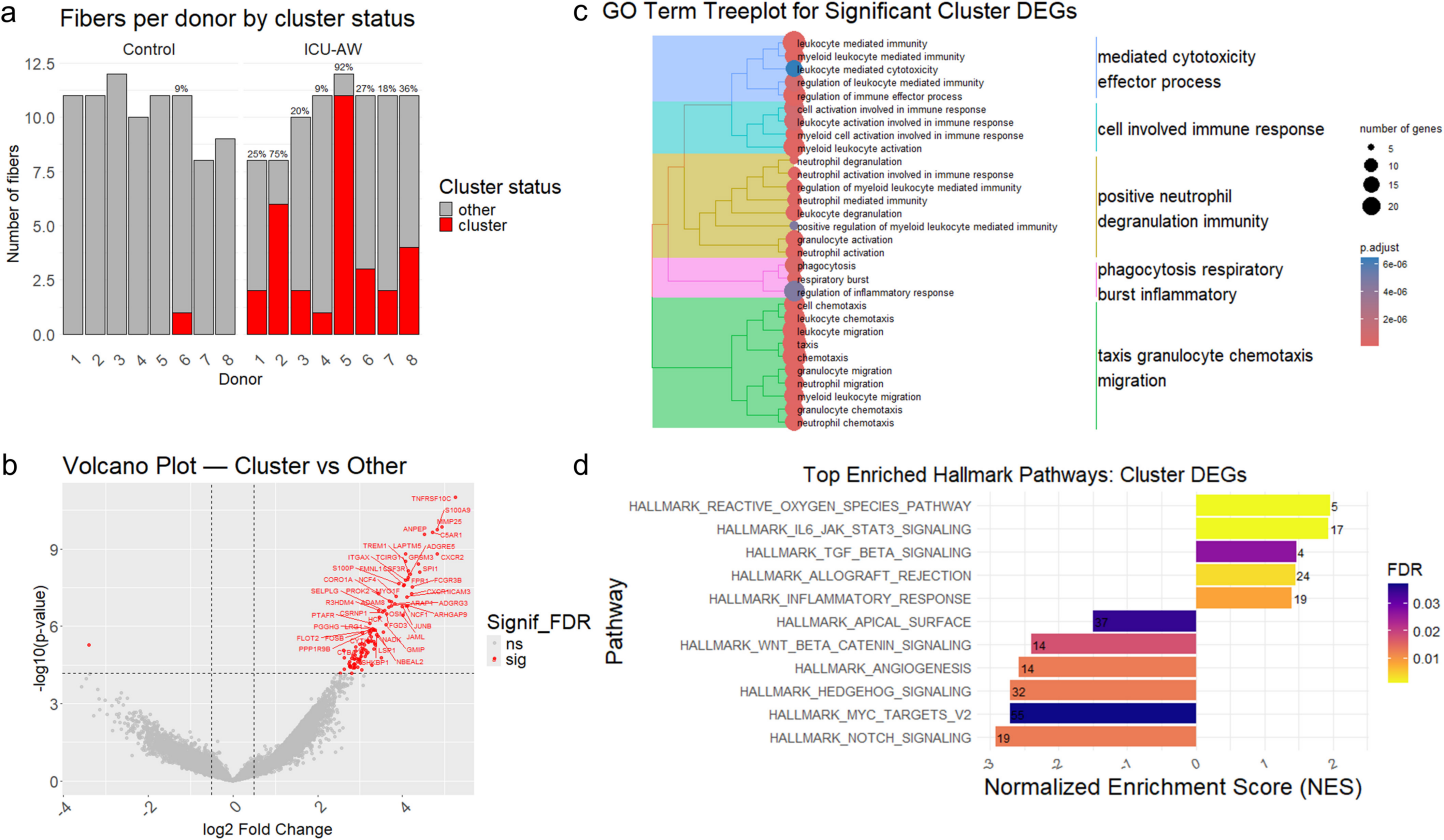
Cluster‐specific composition and transcriptional signatures in Single Myofibres. (a) Cluster composition by donor and condition. Each bar shows total number of fibres, with fibres in cluster coloured red and quantified in percentage. (b) Volcano plot of DEGs between cluster and other. Fibres pseudobulked by donor and cluster status. Red dots indicate significant genes. Top 50 features are labelled. (c) Treeplot of top 30 enriched GO terms for cluster DEGs. This visualisation shows clustering among biologically related GO categories. (d) Hallmark pathway enrichment analysis for cluster DEGs. The significantly enriched pathways are ranked by normalised enrichment score (NES). Bar length represents the NES, and bar colour indicates the FDR‐adjusted *p*‐value. Numbers adjacent to each bar indicate the leading‐edge gene count.

To characterise the ‘Inflammatory’ cluster transcriptional phenotype, differential expression analysis was performed comparing cluster‐assigned versus non‐cluster fibres, aggregated by donor (Figure 3b). This identified 108 DEGs, of which 107 were upregulated in cluster fibres. Gene Ontology (GO) enrichment analysis of identified DEGs revealed strong enrichment for immune‐related processes including mediated cytotoxicity, immune cell activation, neutrophil degranulation, respiratory burst and granulocyte chemotaxis (Figure 3c). Hallmark pathway analysis confirmed significant upregulation of reactive oxygen species, cytokine signalling and immune activation pathways, with corresponding downregulation of developmental and proliferative pathways (WNT/β‐catenin signalling, angiogenesis, Hedgehog signalling, MYC targets, Notch signalling) (Figure 3d). Collectively, these findings reveal a conserved, disease‐enriched transcriptional state mainly defined by inflammatory and chemotactic gene programmes.

### 3.3 Proteomic Profiling Reveals a Coordinated Shift Toward Intracellular Maintenance and Away From Membrane Signalling

Comparable to transcriptomics, direct condition‐level donor comparison revealed no genome‐wide proteomic differences (Figure S4). Filtering out donors with less than 3 fibres maintains no condition‐level proteomic differences (Figure S4). Extending the ‘Inflammatory’ cluster assignment from the transcriptomic analysis, pseudobulk differential expression analysis on the proteomic dataset identified 277 differentially expressed proteins (DEPs) in cluster‐assigned fibres, with 199 upregulated and 78 downregulated compared to fibres outside the ‘Inflammatory’ cluster (Figure 4a). GO enrichment analysis of the 199 upregulated DEPs revealed strong enrichment for mitochondrial alterations, translational machinery, ribosomal biogenesis and wound repair/matrix remodelling (Figure 4b). The 78 downregulated DEPs showed enrichment for membrane‐associated processes and surface protein localisation (Figure 4c). Notably, these proteomic annotations differ from the transcriptomic GO terms (immune activation, stress response), suggesting potential post‐transcriptional or post‐translational control mechanisms shaping the protein‐level phenotype.

**FIGURE 4:**
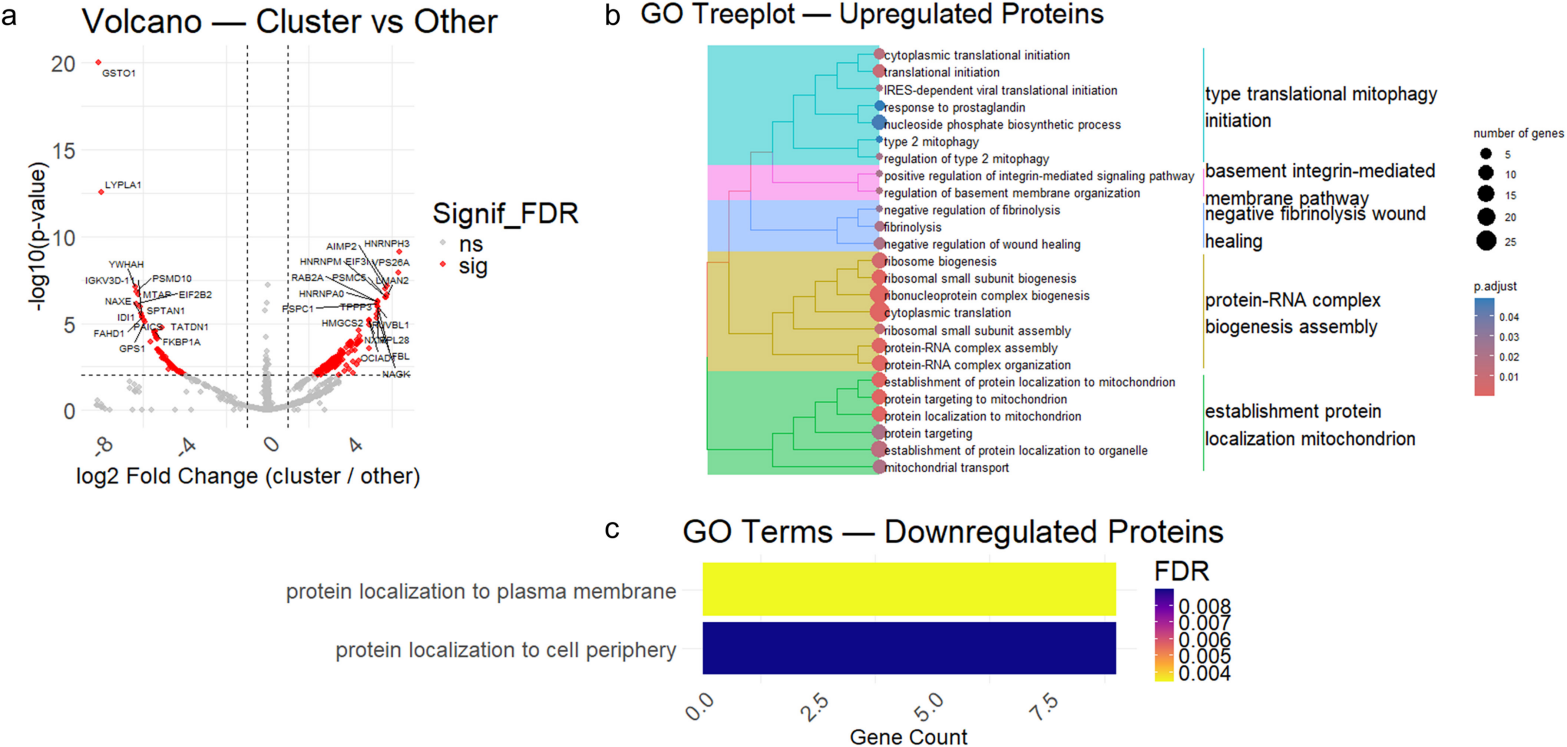
Cluster‐specific proteomic signatures in single myofibres. (a) Volcano plot of DEPs between cluster and other. Fibres pseudobulked by donor and cluster status. Red dots indicate significant proteins. (b) Upregulated GO terms for cluster DEPs. Treeplot with edges represents similarity between terms. (c) Barplot of downregulated proteins, with fill showing FDR‐adjusted *p*‐value.

### 3.4 RNA‐Protein Integration Identifies 19 Concordantly Altered Features Linking Inflammation to Intracellular Remodelling

To assess concordance between transcriptomic and proteomic changes, the datasets were intersected based on shared gene symbols (Figure S5). Directional agreement was assessed using fold changes; 19 features showed significant alterations in both datasets (Figure 5, Table S2). Concordantly upregulated changes included ACTN4, MYL9, VTN, H2AC4, RPS9, CTSG, TPSB2, S100A11 and CDSN. These proteins regulate cytoskeletal anchoring and dynamics (ACTN4, VTN, S100A11), myosin ATPase activity (MYL9), translational control (H2AC4, RPS9) and immune effector functions (CTSG, TPSB2, CDSN). Concordantly downregulated changes included only IDI1, involved in lipid metabolism and membrane biogenesis. Discordant regulations included AUH and NCK2; these showed decreased mRNA but increased protein levels, consistent with selective protein stabilisation. Discordant regulations also included BOLA1, PRR33, SUN2, APOA2, FLOT2, IGKV4‐1 and IGKV3D‐11; these exhibited increased mRNA but decreased protein levels, suggesting enhanced degradation or secretion. Overall, these 19 overlapping features link the inflammatory transcriptional landscape (immune activation, cytotoxicity, ROS) to the proteomic shift toward mitochondrial and translational machinery, revealing a coordinated stress‐adaptation programme with selective post‐translational control.

**FIGURE 5:**
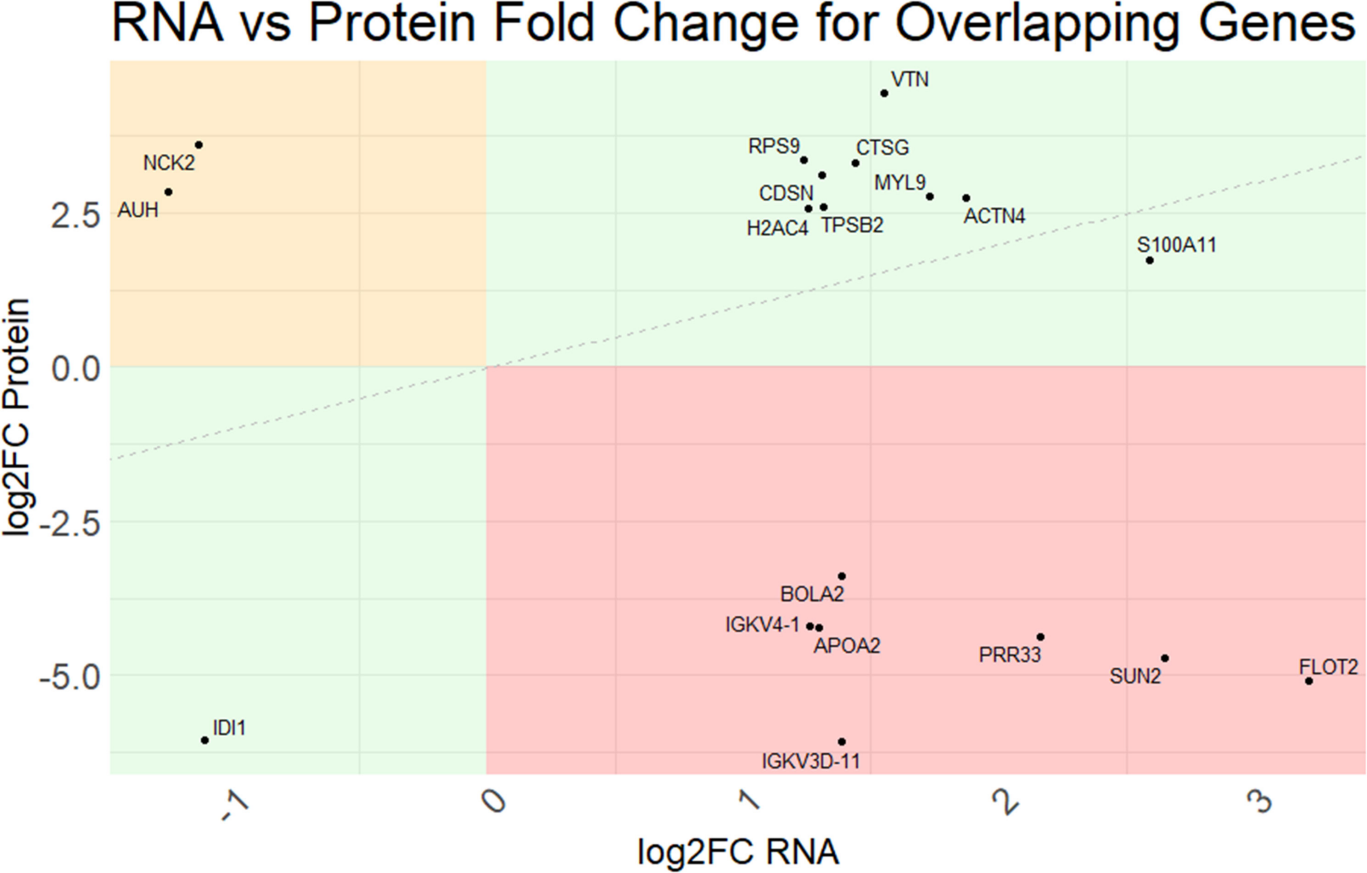
Concordance of RNA and protein fold changes for overlapping genes. Each point represents a feature significantly differentially expressed at both the transcript and protein level. Green = coordinate changes, orange = RNA down/protein up, red = RNA up/protein down. Dashed line represents a 1:1 relationship between RNA and protein fold changes.

### 3.5 Inflammatory‐Clustered Myofibres Display Prolonged ATP Turnover Time for the Myosin Super‐Relaxed State

To assess functional consequences of the ‘Inflammatory’ cluster molecular phenotype, myosin ATP turnover kinetics were measured and correlated with the cluster‐specific molecular features. For each fibre, a composite RNA–protein expression score was calculated as the average z‐score‐normalised expression across the 19 concordantly altered features. This score was tested for correlation with functional measurements: theoretical ATP consumption (Figure 6a), DRX percentage (P1, Figure 6b), SRX percentage (P2, Figure 6c), DRX time constant (T1, Figure 6d) and SRX time constant (T2, Figure 6e).

**FIGURE 6:**
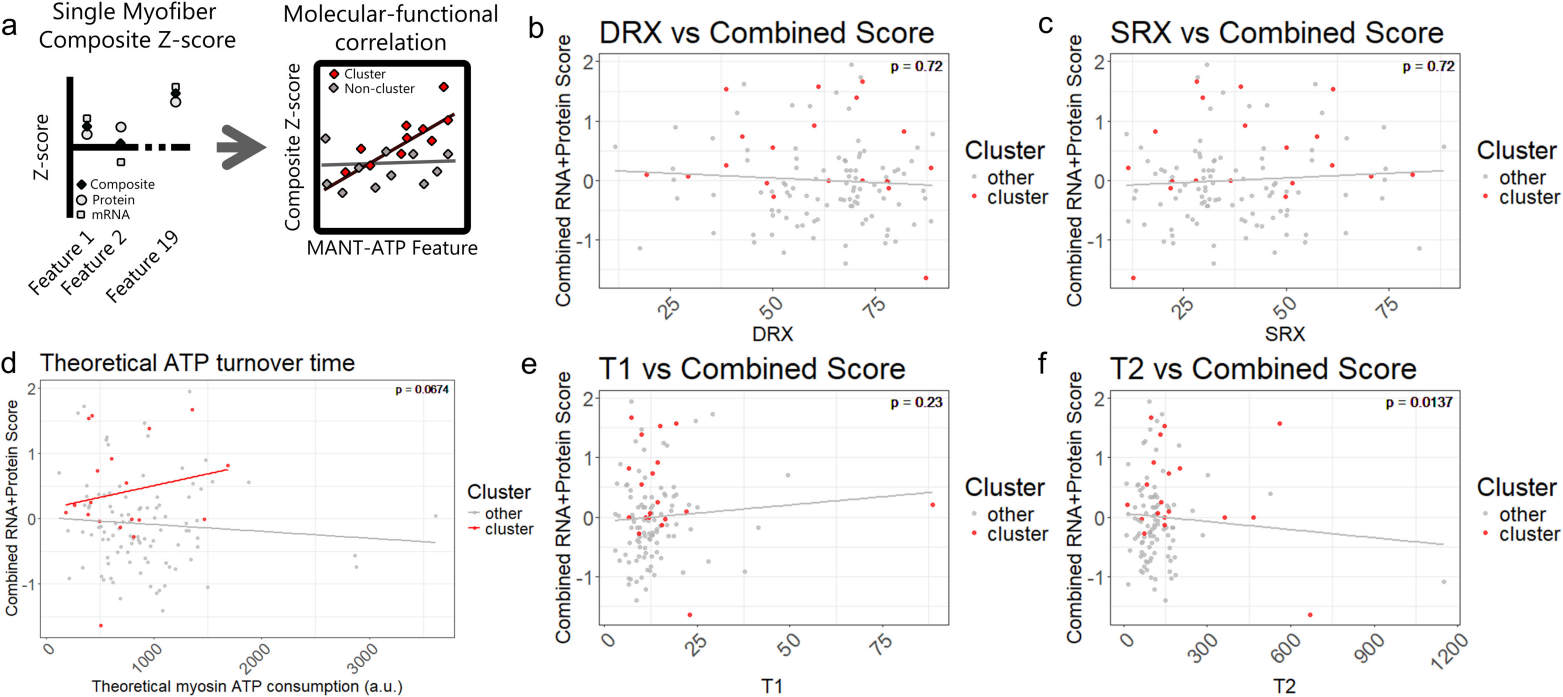
Relationship between functional fibre metrics and overlapping features. (a) Illustration of how per‐fibre expression scores were computed with overlapping RNA–protein differentially expressed features. Scores were Z‐transformed independently for RNA and protein and then averaged to create a score for each fibre. Wilcoxon rank‐sum tests were performed to compare distribution of metric and average cluster/other score (*p*‐value displayed per panel). Linear smooth is illustrative (b, c) Percentage of resting myosin heads in DRX/SRX conformation correlated to integrated expression score. (d) Theoretical ATP turnover time for resting myosin heads correlated to integrated expression score. (e, f) ATP turnover time for myosin heads in DRX/SRX conformation correlated to integrated expression score.

Fibres within the ‘Inflammatory’ cluster showed significantly prolonged ATP turnover time in the SRX state (T2) compared to non‐cluster fibres (Wilcoxon rank‐sum test, *p* < 0.05). There was a significant difference in cluster composite score for T2 (*p* < 0.01), indicating that the molecular features defining the cluster are functionally correlated to altered myosin energetics. Other kinetic parameters (T1, P1, P2, and overall ATP consumption) did not differ significantly between cluster and non‐cluster fibres. This selective prolongation of SRX‐state ATP turnover time indicates reduced basal ATPase activity in resting myosin molecules, consistent with bioenergetic adaptation to the inflammatory and stress‐associated environment. The strong correlation between the multi‐omic cluster score and functional measurements aligns with the molecular phenotype and establishes a link between transcriptomic/proteomic alterations and energy dynamics of the contractile apparatus, uniquely revealed with our workflow.

## 4 Discussion

The complexity of the muscle microenvironment requires coordinated regulation across multiple cellular and molecular layers to maintain contractile function and metabolic homeostasis. Critical illness represents an acute systemic insult that disrupts this coordination, leading to rapid and profound muscle dysfunction known as ICU‐AW [1, 2]. Although numerous molecular profiling studies have characterised skeletal muscle changes in critical illness and ICU‐AW [10, 11, 18, 20, 21, 22, 23, 24, 29], integration of multi‐omic data with direct functional measurements at the level of individual cells remains limited. Previous bulk muscle analyses have shown decreased expression of MyHC isoforms [11, 23, 29], increase in oxidative stress [21, 22, 24] and activation of inflammatory pathways, but do not characterise fibre‐specific changes. Building upon our recent developments [23, 25], this study is a proof‐of‐concept suggesting a novel integrated approach that combines transcriptomic, proteomic and functional myosin measurements from the same single myofibre to identify a disease‐associated fibre state in ICU‐AW. The key innovation is the direct cellular context in which molecular alterations are measured and correlated through functional assessment, enabling interpretation of how coordinated programmes impact contractile performance.

### 4.1 A Conserved Disease‐Associated Myofibre State Emerges Despite Heterogeneity

Despite substantial heterogeneity in both ICU‐AW and control populations (reflecting variation in age, sex, underlying conditions and disease severity), a reproducible myofibre phenotype emerged within the ICU‐AW cohort. This ‘Inflammatory’ cluster phenotype was characterised by distinct transcriptional and proteomic signatures not present in control fibres. The conserved state is present in all ICU‐AW donors and is not explained by myosin heavy chain composition. This suggests that critical illness induces a fibre‐specific molecular programme that, while not uniformly affecting all myofibres, represents a consistent tissue response to systemic stress.

The transcriptional profile of cluster‐assigned fibres emphasised inflammatory and chemotactic processes, indicating either a direct stress response or possible immune cell infiltration or interaction. Concurrently, the proteomic signature revealed a marked shift toward mitochondrial and translational machinery, with reduced membrane‐associated signalling. This dissociation between transcriptomic and proteomic annotations suggests layered post‐transcriptional and post‐translational control.

### 4.2 Multi‐Omic Coordination Reveals Intracellular Prioritisation Under Stress

The identification of 19 concordantly altered features bridges the transcriptomic and proteomic layers to provide mechanistic insight. Firstly, a cytoskeletal and contractile remodelling is suggested by upregulation of ACTN4 and MYL9. This reflects altered actin anchoring and myosin ATPase modulation. ACTN4 overexpression destabilises sarcomeres and induces a structural remodelling phenotype consistent with stress adaptation [30]. Concurrent upregulation of VTN and S100A11 suggests enhanced cell adhesion and cytoskeletal plasticity [31, 32]. Secondly, intracellular maintenance and mitochondrial changes can be linked to increased H2AC4 and RPS9. This indicates heightened translational activity, while downregulation of IDI1 reflects a shift away from lipid synthesis and membrane biogenesis, collectively supporting prioritisation of intracellular repair machinery over extracellular communication. Thirdly, an immune effector integration may occur through elevated CTSG, TPSB2 and aberrant CDSN expression (a glycoprotein not normally expressed in skeletal muscle). This suggests either infiltrating immune cells or myofibre‐intrinsic immune activation, possibly reflecting crosstalk with circulating cytokines or invading leukocytes. Fourthly and finally, our findings indicate post‐translational control through discordant features. AUH and NCK2 are increased despite reduced mRNA, suggesting selective protein stabilisation. AUH dysregulation has been linked to mitochondrial defects and reduced stability of mature RNA transcripts [33], while NCK2 regulates actin cytoskeleton organisation and cell migration [34]. Discordance in elevated mRNA with reduced protein suggests selective degradation of BOLA1, PRR33 and SUN2. PRR33, in particular, is implicated in cytoskeleton–mitochondria organisation, and its degradation reduces myofibre size and strength [35]. This selective protein turnover suggests quality control and resource allocation mechanisms tuned toward mitochondrial maintenance and away from full‐fibre growth and membrane‐associated functions.

### 4.3 Altered Myosin Super‐Relaxed State Dynamics Link Molecular Adaptation to Energetic Capacity

The functional finding of prolonged ATP turnover time for the SRX state connects the multi‐omic phenotype to myosin energetics. The SRX state represents the resting conformation with minimal basal ATPase activity, and prolonged residence in this state reduces energetic cost during periods of inactivity. However, a shift toward SRX can also reflect impaired transition back to the active state, reducing maximal contractile capacity. The strong correlation between the composite multi‐omic score and T2 (SRX time constant) validates the molecular cluster and suggests that stress‐induced transcriptional and translational remodelling fundamentally alters myosin dynamics. This energetic shift aligns with the coordinated upregulation of MYL9 and ACTN4, known regulators of myosin ATPase activity and contractile dynamics. The proteomic emphasis on mitochondrial and translational machinery indicates a cell‐autonomous bioenergetic adaptation (shifting resources toward maintenance and energy provision) at the expense of sustained contractile capacity. This may represent a compensatory cellular response to acute energy scarcity: by reducing basal ATP demand and prioritising mitochondrial function, the stressed fibre sacrifices immediate contractile power for energetic survival.

### 4.4 Methodological Implications and Biological Significance

By integrating transcriptomic, proteomic and functional measurements from individual myofibres, this work supports an approach that reveals cellular heterogeneity and functional adaptation beyond that which traditional bulk tissue analysis or single‐cell sequencing technologies, can capture. Single‐fibre transcriptomics or proteomics alone might identify individual molecular changes; only the integrated approach can reveal how these changes coordinate to reshape cellular function. This integrative framework is broadly applicable to complex disease states in which cellular heterogeneity obscures population‐level signals. The single‐fibre approach also mitigates a major confounding factor in bulk muscle studies: the mixture of fibre types, developmental states and cell types within tissue. By measuring the same fibre across molecular and functional dimensions, the precision and interpretability of findings are substantially enhanced. This approach contextualises molecular signals within their native cellular environment and reveals how adaptive programmes couple to molecular and contractile layers.

### 4.5 Clinical Implications and Therapeutic Opportunities

The identification of a conserved disease‐associated fibre state may have important clinical implications. Muscle dysfunction in critical illness is a major determinant of morbidity and mortality, yet current therapeutic options are limited. The characterisation of a fibre‐specific molecular and functional phenotype opens new avenues for intervention. In finding a link between energetic shift and structural remodelling, this study supports a role for interventions that increase the availability of energy. Importantly, muscle dysfunction in critical illness may reflect a state of bioenergetic failure, in which impaired energy supply and/or utilisation leads to early functional impairment that can precede overt structural damage. This framework further supports targeting mitochondrial dysfunction and inflammatory stress responses to preserve excitability and contractile performance [15].

Within this framework, several of the concordantly and discordantly regulated features identified here suggest specific testable targets. Given their concordant up‐regulation at both transcript and protein level, ACTN4 and MYL9 are potential immunohistochemical biomarkers of contractile remodelling in ICU‐AW biopsies, though their potential as circulating biomarkers remains unclear. Because PRR33 loss is related to reduced fibre size and strength [35], pathways preserving its stability represent a more speculative route for intervention. Furthermore, the multi‐omic integration provides a framework for patient stratification: fibres or patients with higher abundance of the ‘Inflammatory’ cluster phenotype might represent a distinct therapeutic target population.

### 4.6 Limitations and Future Directions

Several limitations should be addressed in future studies. First, the sample size, while appropriate for a proof‐of‐concept, limits the bioinformatic models and approaches that could be used. Future studies incorporating larger cohorts, with balanced sex, age and geographical distributions, will be essential for validating the conserved fibre state and establishing its prevalence and clinical relevance across different ICU populations and disease etiologies. As such, the findings are preliminary and should be interpreted with appropriate caution. Second, this study measures ATP turnover kinetics but future work incorporating force measurements would clarify the functional impact of altered myosin dynamics on contractile performance. Third, extended proteomic analyses could reveal post‐translational modifications (phosphorylation, acetylation, ubiquitination) that underlie the selective protein stabilisation and degradation observed here.

## 5 Conclusions

This study suggests that in critically ill patients, ICU‐AW induces a conserved, fibre‐specific molecular and functional phenotype characterised by inflammatory signalling, intracellular remodelling and altered myosin energetics. By directly correlating multi‐omic alterations to myosin dynamics at the level of individual myofibres, this work provides a proof‐of‐concept for a broadly applicable integrative framework for dissecting cellular heterogeneity and functional adaptation in complex human diseases. The identification of coordinated transcriptomic and proteomic programmes, validated through functional measurement, suggests that targeting this fibre state may offer new therapeutic opportunities in ICU‐acquired dysfunction and related muscle disorders.

## Funding

This work was supported by Lundbeck Foundation R434‐2023‐311.

## Conflicts of Interest

The authors declare no conflicts of interest.

## Supporting information


**Image S1** Representative bright field (63×) of myofibre and sarcomeric structure (a) Control myofibre. (b) ICU‐AW myofibre.
**Table S1:** Workflow and final fibre inclusion information.
**Table S2:** Gene annotations for overlapping RNA–protein differentially expressed features. Manually annotated functional descriptions for features exhibiting concordant differential regulation at both the transcriptomic and proteomic level. Annotations were compiled from NCBI gene summaries.
**Figure S1:** Fibre‐ and donor‐level PCA for RNA‐seq and proteomic data. (a) Fibre‐level PCA on Harmony batch corrected transcriptomic data. (b) Donor‐level PCA on pseudobulk transcriptomic data. (c) Fibre‐level PCA on normalised, scaled and imputed proteomic data. (d) Donor‐level PCA on pseudobulk proteomic data.
**Figure S2:** Jaccard similarity heatmap showing pairwise gene set overlap among MSigDB Hallmark pathways. The Jaccard index reflects the proportion of shared genes between two pathways relative to the size of their union.
**Figure S3:** Cluster composition. (a) Stacked barplot coloured by fibre type (based on myosin heavy chain expression), showing composition of cluster group compared to other fibres. (b) Feature counts per fibre, showing comparable coverage of cluster fibres to other fibres.
**Figure S4:** DEPs between ICU‐AW and control samples. (a) Volcano plot with fibres aggregated by donor. Grey dots indicate non‐significant proteins. (b) Number of fibres per donor; donors with less than three fibres are highlighted. (c) Volcano plot with fibres aggregated for donors with at least three fibres. Grey dots indicate non‐significant proteins.
**Figure S5:** Concordance of RNA and protein fold changes for all features. Each point represents the fold changes of a feature differentially expressed at both the transcript and protein level. Red points are features significantly changed in both RNA and protein datasets. Dashed line represents a 1:1 relationship between RNA and protein fold changes.

## Data Availability

RNA‐sequencing data have been deposited in the European Genome‐phenome Archive under study ID EGAS50000001649. The mass spectrometry proteomics data have been deposited to the ProteomeXchange Consortium via the PRIDE partner repository with the dataset identifier PXD073993. All bioinformatics code and analysis scripts are available on GitHub (https://github.com/amwinant/ICUAW‐SFMO‐pilot.git).
