# Supplementary material for: Integrated Single‐Myofibre Multi‐Omics Correlates Inflammatory State to Myosin Energetics in ICU‐Acquired Weakness
Source: J Cachexia Sarcopenia Muscle. 2026 Oct 19;17(5):e70407. doi: 10.1002/jcsm.70407 (PMC13680048; doi:10.1002/jcsm.70407)
Supplement: Supplementary file 1 [file JCSM-17-e70407-s001.docx]

Supplementary file for:

**Integrated single-myofiber multi-omics correlates inflammatory state to myosin energetics in ICU-acquired weakness**

Alexandra M. Winant, Roger Moreno-Justicia, Lucia Paolini, Wout J. Claassen, Coen A.C. Ottenheijm, Atul S. Deshmukh, Stefano Cattaneo, Simone Piva, Nicola Latronico, Robert A.E. Seaborne, Julien Ochala

This document includes:

- Methodological details
- Image S1
- Tables S1 and S2
- Figures S1 – S5

**Methodological details**

Tissue Collection and Storage

Human skeletal muscle tissue was dissected from quadriceps femoris. The muscle samples harvested from patients hospitalized in ICU were obtained with a surgical biopsy, 8-10 days after ICU admission. The skin was prepped with antiseptic Clorhexidin 2% solution and draped. A longitudinal 4 cm midline incision was performed at the middle third of the thigh. The quadriceps muscular fascia was reached through the subcutaneous tissue and longitudinally incised. Hemostasis was obtained in every case by compression. The quadriceps fascia and the subcutaneous tissue were sutured with 3-0 polyglactin 910 braided adsorbable suture; the skin was sutured with 4-0 polyglactin 910 braided adsorbable suture. A compressive dressing was applied at the end of the procedure. The samples obtained from patients in the Orthopedic Ward were obtained during total hip replacement performed for hip arthritis through a direct anterior approach. As soon as the biopsy was removed from the patient, it was immediately placed into a polypropylene tube filled with 25 ml cold (+4°C) NaCl 0.9% solution and washed twice. Skeletal muscle tissue was, then, moved to a new tube and snap-frozen in liquid nitrogen. Samples were stored at -80°C.

Sample preparation for Proteomics Analyses

Ice-cold 100% acetone in 100 mM Tris-HCl (pH 8.5) was incubated with proteins overnight. Precipitation was performed with sequential centrifugation and washing in 100% acetone/100 mM Tris buffer. Acetone was evaporated from the samples using a SpeedVac concentrator. Pellets were resuspended in 10 μL of digestion buffer (composition: 1% sodium deoxycholate, 10 mM DTT, 40 mM chloroacetamide, 50 mM Tris-HCl pH 8.5) and boiled at 95°C with shaking for 10 minutes. Samples were briefly centrifuged, then sonicated for 15 minutes in a water bath sonicator.

Protein digestion was performed overnight at 37°C using a mix of trypsin and Lys-C (1:100 and 1:500 enzyme to protein ratios, respectively). The next day, peptides were desalted using in-house-prepared Stage-Tips packed with Styrene divinylbenzene-reverse phase sulfonate (SDB-RPS) disks.

Digested samples were transferred onto tips and centrifuged until complete loading. Tips were first washed with 1% trifluoroacetic acid (TFA) in isopropanol followed by a wash with 0.2% TFA in water. The resulting desalted peptides were eluted with 80% acetonitrile and 1% ammonia solution. Samples were dried in a SpeedVac and resuspended in A* buffer (5% acetonitrile, 0.1% TFA). Samples were transferred to Evotips according to the manufacturer's instructions prior to LC-MS/MS analysis.

The LC instrumentation consisted of an Evosep One HPLC system (Evosep, Odense, Denmark) coupled by electrospray ionisation to a timsTOF HT SCP mass spectrometer (Bruker, MA, USA). Peptide separation was achieved on an 8 cm column (150 µm inner diameter) packed with 1.5 µm C18 particles. Chromatography was conducted using the “60 samples per day” gradient method, with ionisation via a CaptiveSpray2 source and a 20 µm emitter for MS introduction. Single muscle fiber peptide samples were analysed using DIA-PASEF, employing an acquisition of 100-1700 m/z range. The shot-gradient diaPASEF method was used, including 8 diaPASEF scans with three 25 Da windows per ramp, a total mass range of 475-1000 Da, and a mobility range of 1.30-0.85 1/K0. The collision energy was decreased linearly from 59 eV at 1/K0 = 1.6 to 20 eV at 1/K0 = 0.6 Vs cm-2. Both accumulation time and PASEF ramp time were set to 100 ms, with a total cycle time of 0.95 s across 8 DIA-PASEF scans.

Downstream Transcriptomic Analysis

Quality control filtering removed fibers with gene counts or feature abundance ±3 median absolute deviation (MAD) from the median. Fibers meeting QC criteria were retained for subsequent analysis (final n = 165 fibers). Library-size normalization was performed using LogNormcounts (Scran version 1.34.0).^1^ Batch effects, namely number of PCR cycles and sequencing lane, were corrected using Harmony on the default 50 PCs.^2^

**Differential Expression Analysis**: Differential expression analysis was conducted using a pseudobulk approach to account for the nested donor structure. Raw counts were aggregated across all fibers within each donor-condition combination. Pseudobulk counts were normalized using the trimmed mean of M-values (TMM, edgeR version 4.4.2)^3^ method and analyzed using limma–voom (version 3.62.2).^4^ Count data were converted to log₂ counts per million (CPM), and linear regression modeling with empirical Bayes moderation was applied. Differentially expressed genes were ranked by false discovery rate (FDR) using the Benjamini-Hochberg method; significance threshold was FDR < 0.05.

**Gene Set Enrichment Analysis**: GSEA was performed on the ranked DEG list using the fgsea algorithm (version 1.32.4)^5^ with 10,000 permutations. Hallmark gene sets from MSigDB (version 25.1.1)^6^ were tested for enrichment. Normalized enrichment scores (NES) and FDR-adjusted p-values were calculated.

**Cluster Identification and Characterization:** The top 50 DEGs were selected for visualization at the single-fiber level. Expression values were z-score normalized across all fibers. Hierarchical clustering (Euclidean distance, complete linkage) and heatmap visualization were performed using pheatmap (version 1.0.13).^7^ Fibers were assigned to cluster or non-cluster groups based on dendrogram membership.

To characterize the cluster phenotype, differential expression analysis compared cluster-assigned versus non-cluster fibers, aggregated by donor and cluster status. Overexpression gene Ontology (GO) enrichment analysis was performed using the clusterProfiler package (version 4.14.6)^8^ separately for up- and downregulated genes.

Downstream Proteomic Analysis

Fiber types were assigned based on the relative abundance of myosin heavy chain (MYH) isoforms measured at the protein level. Log2-normalized and imputed protein intensities for MYH7, MYH2, and MYH1 were extracted for each individual fiber and transformed back to linear scale. For each fiber, the relative contribution of each MYH isoform was calculated as a percentage of the total MYH signal (MYH7 + MYH2 + MYH1). Fibers were then ordered from highest to lowest relative expression for each MYH isoform independently. Data-driven thresholds for dominant isoform expression were determined using a bottom-knee detection approach based on the point of maximal curvature in the ordered distribution. Fibers not meeting criteria for a dominant or hybrid classification were labeled as other hybrid fibers.^9^

Differential protein expression analysis was performed using the same pseudobulk approach as the transcriptomic analysis. GO enrichment analysis was conducted separately for up- and downregulated proteins.

Methodological references

1.	Lun, A. T. L., McCarthy, D. J. & Marioni, J. C. A step-by-step workflow for low-level analysis of single-cell RNA-seq data with Bioconductor. *F1000Research* **5**, 2122 (2016).

2.	Lytal, N., Ran, D. & An, L. Normalization Methods on Single-Cell RNA-seq Data: An Empirical Survey. *Front. Genet.* **11**, (2020).

3.	Chen, Y., Chen, L., Lun, A. T. L., Baldoni, P. L. & Smyth, G. K. edgeR v4: powerful differential analysis of sequencing data with expanded functionality and improved support for small counts and larger datasets. *Nucleic Acids Res.* **53**, gkaf018 (2025).

4.	Ritchie, M. E. *et al.* limma powers differential expression analyses for RNA-sequencing and microarray studies. *Nucleic Acids Res.* **43**, e47–e47 (2015).

5.	Korotkevich, G. *et al.* Fast gene set enrichment analysis. Preprint at https://doi.org/10.1101/060012 (2016).

6.	Dolgalev, I. msigdbr: MSigDB Gene Sets for Multiple Organisms in a Tidy Data Format. 25.1.1 https://doi.org/10.32614/CRAN.package.msigdbr (2018).

7.	Kolde, R. pheatmap: Pretty Heatmaps. 1.0.13 https://doi.org/10.32614/CRAN.package.pheatmap (2010).

8.	Xu, S. *et al.* Using clusterProfiler to characterize multiomics data. *Nat. Protoc.* **19**, 3292–3320 (2024).

9.	Moreno-Justicia, R. *et al.* Human skeletal muscle fiber heterogeneity beyond myosin heavy chains. *Nat. Commun.* **16**, 1764 (2025).

**Image S1. Representative bright field (63x) of myofiber and sarcomeric structure** a) Control myofiber. b) ICU-AW myofiber

**Table S1.** **Workflow and final fiber inclusion information.**

| Donor ID | Condition | Age | Fibers in functional analysis | Fibers in transcriptomic analysis | Fibers in proteomic analysis | Overlapping fibers for integration analysis |
| --- | --- | --- | --- | --- | --- | --- |
| 1 | Control | 66 | 12 | 11 | 10 | 10 |
| 2 | Control | / | 12 | 11 | 10 | 10 |
| 3 | Control | 62 | 12 | 12 | 6 | 6 |
| 4 | Control | 43 | 12 | 10 | 10 | 8 |
| 5 | Control | 53 | 12 | 19 | 10 | 10 |
| 6 | Control | 82 | 12 | 11 | 7 | 6 |
| 7 | Control | 66 | 12 | 8 | 7 | 6 |
| 8 | Control | 73 | 12 | 9 | 7 | 7 |
| 1 | ICU-AW | 19 | 12 | 8 | 1 | 1 |
| 2 | ICU-AW | 37 | 12 | 8 | 3 | 2 |
| 3 | ICU-AW | 71 | 12 | 10 | 9 | 8 |
| 4 | ICU-AW | 81 | 12 | 11 | 11 | 11 |
| 5 | ICU-AW | 30 | 12 | 12 | 11 | 11 |
| 6 | ICU-AW | 84 | 12 | 11 | 10 | 9 |
| 7 | ICU-AW | 88 | 12 | 11 | 7 | 7 |
| 8 | ICU-AW | 66 | 12 | 11 | 5 | 5 |

**Table S2. Gene annotations for overlapping RNA–protein differentially expressed features.** Manually annotated functional descriptions for features exhibiting concordant differential regulation at both the transcriptomic and proteomic level. Annotations were compiled from NCBI gene summaries.

| Gene | Full name | Function summary |
| --- | --- | --- |
| ACTN4 | Actinin Alpha 4 | Helps anchor the myofibrillar actin filaments |
| MYL9 | Myosin light chain 9 | Regulates muscle contraction by modulating the ATPase activity of myosin heads |
| IDI1 | Isopentenyl-diphosphate delta isomerase 1 | Peroxisomally-located, catalyzes substrate for ultimate synthesis of cholesterol |
| VTN | Vitronectin | Promotes cell adhesion, migration, ECM degradation |
| H2AC4 | H2A clustered histone 4 | Replication-dependent histone that is a member of the histone H2A family |
| RPS9 | Ribosomal protein S9 | Ribosomal protein that is a component of the 40S subunit |
| CTSG | Cathespin G | Component of azurophil granules of neutrophilic polymorphonuclear leukocytes |
| TPSB2 | Tryptase beta 2 | Trypsin-like serine protease, main isoenzymes expressed in mast cells |
| CDSN | Corneodesmosin | Glycoprotein typically localized to human epidermis |
| S100A11 | S100 calcium binding protein A11 | Motility, invasion, and tubulin polymerization |
| AUH | AU RNA binding methylglutaconyl-CoA hydratase | Involved in RNA degradation and deadenylation, mitochondrial protein synthesis |
| NCK2 | NCK adaptor protein 2 | Cytoskeletal reorganization |
| BOLA1 | bolA family member 1 | Cell redox homeostasis, mitochondrial protein |
| IGKV4-1 | Immunoglobulin kappa variable 4-1 | Antigen binding activity, immune response |
| APOA2 | Apolipoprotein A2 | Second most abundant protein of the high density lipoprotein particles |
| PRR33 | Proline rich 33 | Wound healing |
| SUN2 | Sad1 and UNC84 domain containing 2 | Inner nuclear membrane, connection between nuclear lamina and cytoskeleton |
| FLOT2 | Flotillin 2 | Vesicular trafficking and signal transduction |
| IGKV3D-11 | Immunoglobulin kappa variable 3D-11 | Immune response, located in extracellular region and plasma membrane |

**Figure S1. Fiber- and donor-level PCA for RNA-seq and proteomic data.** a) Fiber-level PCA on Harmony batch corrected transcriptomic data. b) Donor-level PCA on pseudobulk transcriptomic data. c) Fiber-level PCA on normalized, scaled, and imputed proteomic data. d) Donor-level PCA on pseudobulk proteomic data.

**Figure S2. Jaccard similarity heatmap showing pairwise gene set overlap among MSigDB Hallmark pathways.** The Jaccard index reflects the proportion of shared genes between two pathways relative to the size of their union.

**Figure S3. Cluster composition.** a) Stacked barplot colored by fiber type (based on myosin heavy chain expression), showing composition of cluster group compared to other fibers. b) Feature counts per fiber, showing comparable coverage of cluster fibers to other fibers.

**Figure S4. DEPs between ICU-AW and control samples.** a) Volcano plot with fibers aggregated by donor. Gray dots indicate non-significant proteins. b) Number of fibers per donor; donors with less than 3 fibers are highlighted. c) Volcano plot with fibers aggregated for donors with at least 3 fibers. Gray dots indicate non-significant proteins.

**Figure S5. Concordance of RNA and protein fold changes for all features.** Each point represents the fold changes of a feature differentially expressed at both the transcript and protein level. Red points are features significantly changed in both RNA and protein datasets. Dashed line represents a 1:1 relationship between RNA and protein fold changes.
